# Toxicological evaluation of a saponin-rich standardized extract of fenugreek seeds (FenuSMART^®^): Acute, sub-chronic and genotoxicity studies

**DOI:** 10.1016/j.toxrep.2018.10.008

**Published:** 2018-10-09

**Authors:** D. Sureshkumar, Shamshad Begum, N.M. Johannah, Balu Maliakel, I.M. Krishnakumar

**Affiliations:** a CARe-KERALAM Ltd., Kinfra Small Industries Park, Thrissur, 680309, Kerala, India; b University of Agricultural Sciences, Bangalore, 560024, Karnataka, India; c R&D Centre, Akay Flavours & Aromatics Ltd., Cochin, 683561, Kerala, India

**Keywords:** Fenugreek extract, *Trigonella foenum graecum*, Phytoestrogen, Menopause, Subchronic toxicity, Genotoxicity

## Abstract

The present study investigated the safety of a saponin-rich standardized extract of fenugreek seeds (FenuSMART^®^; FHE), that has been clinically shown to be effective in ameliorating the postmenopausal discomforts and establishing hormonal balance. The safety was assessed by oral acute (2500 mg/kg b. wt. for 14 days) and subchronic (250, 500 and 1000 mg/kg b. wt. for 90 days) toxicity studies on Wistar rats and mutagenicity studies employing *Salmonella typhimurium* strains. Administration of FHE did not produce any toxicologically significant changes in clinical/behavioral observations, ophthalmic examinations, body weight, organ weight, feed consumption, urinalysis, hematology and clinical biochemistry parameters when compared to the untreated control group of animals. Highest dose recovery group (1000 mg/kg b. wt.) of animals also showed no mortality or adverse events; with hematological and biochemical parameters at par with those of controls. Terminal autopsy revealed no alterations in relative organ weight or any treatment-related histopathology changes. FHE also showed no mutagenicity upon Ames test employing TA-98, TA-100 and TA-102 *Salmonella typhimurium* strains with or without metabolic activation. Based on the results of the study, the no observed-adverse-effect level (NOAEL) of FHE was determined as 1000 mg/kg b. wt./day, the highest dose tested.

## Highlights

• Safety evaluation of a standardized extract of fenugreek seeds (FenuSMART).
• Acute and subchronic oral toxicity studies of FenuSMART on Wistar rats.
• Mutagenicity study of FenuSMART.

## Graphical abstract

**Figure:**
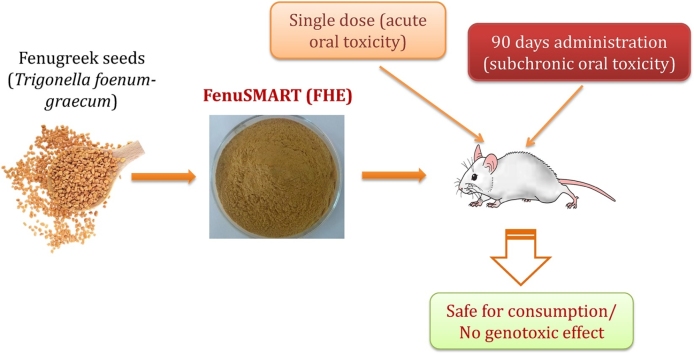

## 1 Introduction

Ever since the beginning of civilization, man has looked at nature not only for the taste and flavor, but also for remedies to heal the body, mind and the soul. The development and application of natural medicines were practicing as the main stream treatment option until 19^th^ century. Spices, a group of aromatic plants popular as food flavors and preservatives, constitute an important class of medicinal plants well-practiced in Indian and Chinese traditional systems of medicine. Evidence based scientific research in the past two to three decades could identify spices as a hidden treasure of phytonutrients capable of delivering significant health protective/maintaining pharmacological effects [1]. Moreover, various natural remedies based on spices were also shown to be multifunctional, pleiotropic and free of side effects as compared to the specific receptor targeted synthetic drugs [2]. Moreover, various molecular techniques in the post-genomic era could prove that the consumption of effective dosage of bioavailable natural antioxidants, anti-inflammatory agents and phytoestrogens, (especially those derived from food components such as fruits, vegetables and spices), can prevent and/or ameliorate the pathogenesis of many chronic diseases [3,4]. Thus, the supplementation of standardized spice extracts as capsules/tablets (Nutraceuticals) has emerged as an alternative and complementary approach to the prevention and management of various disease states.

Among the various popular kitchen spices, the turmeric, ginger, cinnamon, chili, black pepper, clove and fenugreek include the most widely studied spices for their medicinal and nutritional properties [5]. Though various extracts of these spices have been subjected to detailed investigations on their pharmacological effects, only fenugreek (*Trigonella Foenum Graecum*) was shown to possess phytoestrogenic activities [6]. Fenugreek is an annual plant belonging to the family *Fabaceae,* and widely cultivated in India, with an annual production of around 121,775 tons of seeds from an area of 96,304 ha [7]. It is generally recognized as safe (GRAS) by US Food and Drug Administration (USFDA) and is a popular herb possessing a wide range of medicinal applications [8,9]. Fenugreek seeds were traditionally used to treat diabetes, hyperlipidemia, gastric ulcers, burns and wounds along with a range of women health beneficial effects including galactogogue property [10]. They were identified as rich source of steroidal saponins (protodioscin, diosgenin, yamogenin), polyphenols (choline, luteolin, orientin, quercetin), alkaloids (pyridine, trigonellin), a non-proteinogenic amino acid ‘4-hydroxyisoleucine’ and galactomannans [9,11,12,3]. Fenugreek seeds were clinically evaluated for pre- and post-menopausal discomforts [[13], [14], [15], [16]]. Earlier studies reported the ability of a hydro-ethanolic extract of fenugreek seeds in improving the sexual function of healthy men and women [17,18]. It was also reported that fenugreek extract possess potential neuroprotective action in patients with Parkinson’s disease and demonstrated the safety and beneficial effect when used as an adjuvant to L-Dopa therapy [19]. Other clinically investigated health beneficial effects of fenugreek include blood-sugar management [20] and lipid profile management [21,22].

In the present contribution, safety assessment of a saponin-rich standardized fenugreek husk extract containing protodioscin (> 10% w/w), trigonelline (> 3% w/w) and 4-hydroxyisoleucine (> 3% w/w)] (FenuSMART^®^; *herein after referred to as ‘FHE’*) was reported. Acute (14 days), and sub-chronic (90-days) toxicity studies were carried out along with the Ames test for mutagenicity. Despite the toxicity studies of various extracts of fenugreek seeds [[23], [24], [25]], the significance of the present study lies in the fact that the unique composition of various phytochemicals in ‘FHE’ was shown to possess significant efficacy in ameliorating the postmenopausal discomforts and helps to establish the hormone balance [26].

## 2 Materials and methods

### 2.1 Materials

Dried fenugreek seeds were received from a selected farm in India and were identified by an authenticated botanist. A voucher specimen (AK-FEN-012) was deposited at the Herbarium of M/s Akay Flavours & Aromatics Ltd., Cochin, India. An authentic sample of FHE (Batch No: FHE 01/14), prepared by hydro-ethanolic extraction followed by purification and spray drying, was obtained from the manufacturer along with a detailed certificate of analysis indicating its phytochemical compositions, nutritional composition, microbiological status, and heavy metals along with the pesticide and mycotoxin analysis report. FHE was found to contain 52.6% (w/w) of saponins with 11.4% (w/w) of protodioscin along with 3.4% (w/w) of trigonellin and 3.6% (w/w) of 4-hydroxyisoleucine. HPLC analysis was carried out on a Shimadzu model LC 20 AT, with M20 A photodiode array (PDA) detector (Shimadzu Analytical India Pvt. Ltd., Mumbai, India), fitted with a reverse phase C18 column (250 × 4.6 mm, 3 μm) (Phenomenex, Hyderabad, India). UV/VIS analyses were performed on Varian-Cary 5000 UV-VIS-NIR spectrophotometer (Varian Medical Systems Inc, California, USA). Analytical standards of protodioscin, trigonellin and 4-hydroxyisoleucine were obtained from Chromadex, USA. All solvents of HPLC and analytical grade were obtained from Merck, Mumbai, India.

### 2.2 Animals

Adult male and female Wistar rats aged 8 to 10 weeks (170 ± 20 g) were used for toxicological studies. The animals were procured from Veterinary College, Mannuthy, Kerala, India, and were acclimatized for a period of 14 days in polypropylene cages housed at the animal house facility of CARe-KERALAM research centre, Kerala, India. Each cage contained rats of the same sex with a bedding of husk and were placed in an air-conditioned room at 22 ± 2◦C and relative humidity of 60 ± 5% with 12 h light and dark cycle. The animals were fed with the feed pellets provided by M/s. VRK nutritional solutions, Mumbai, India and water *ad libitum.* Deep bore-well water passed through activated charcoal filter and exposed to ultraviolet rays in aqua guard water filter cum purifier (Eureka Forbes Ltd., Mumbai, India) was provided in plastic water bottles with stainless steel sipper tubes. All animal experiments were carried out in strict accordance with the ethical norms approved by the Institutional Animal Ethics Committee (IAEC) recognized by the Committee for the Purpose of Control and Supervision of Experiments on Animals (CPCSEA), Government of India (Registration No: 1620/PO/RcBi/S/12/CPCSEA).

### 2.3 Toxicity studies

#### 2.3.1 Oral acute toxicity studies (14 days)

Forty rats were divided into four groups, with each group containing five animals per sex having similar weights (170 ± 20 g). Group I was the control (untreated), and Groups II, III and IV were administered with FHE at 0.5, 1.0 and 2.5 g/kg b. wt. respectively. The dose was selected from previous studies [24,25,27]. All the animals were observed for mortality, clinical and behavioral signs for the first 10, 30, 60, 120, 240 and 360 min post dose, and thereafter twice daily for mortality and once daily for clinical signs during the study period of 14 days. The animals were examined particularly for changes in skin, fur, and an occurrence of secretions, excretions and autonomic activity. Followed by daily observation, individual animal body weights were recorded at one day before dosing (day 0), and every day until 14 days. All animals were euthanized at the end of the observation period and subjected to a complete necropsy. As no gross pathological findings were encountered in any of the organs, histopathological examination was not conducted.

#### 2.3.2 90-days repeated dose (subchronic) oral toxicity study

Sixty rats (30 males and 30 females), aged 8–10 weeks, with an average body weight 170 ± 20 g were selected by stratified randomization and then divided into six groups, each consisting of five male and five female, and were fed with FHE for 90 days as follows.

Group I - Control

Group II - Control recovery

Group III - Low dose FHE (250 mg/kg)

Group IV – Mid dose FHE (500 mg/kg)

Group V - High dose FHE (1000 mg/kg)

Group VI - High dose recovery (1000 mg/kg)

FHE was suspended in distilled water (10 ml/kg body weight) and orally administered to the animals using an oral feeding tube in such a way that all the animals received same volume of vehicle. The dose was selected from previous studies [24,25,27]. The control group received equivalent quantity of water orally. Animals have been fasted prior to dosing. Animals were observed for signs and symptoms, behavior alteration, food and water intake, body weight changes and mortality. Body weight, food and water consumption were determined every week for 90 days and expressed for a single cage of five animals. At the end of the 90-day period, the animals, except the recovery group, were fasted overnight and blood samples were collected from the orbital sinus for analyzing the hematological parameters and serum biochemistry. The animals were sacrificed by cervical dislocation under ether anesthesia and necropsy was performed in the presence of a veterinary doctor and examined visibly for any type of abnormalities. Organs (Liver, Heart, Spleen, Lungs and Kidney) were isolated and weighted. The control recovery and high dose recovery group is kept for post observation for 28 days.

#### 2.3.3 Hematological analyses

The total blood was collected by direct heart puncture method into EDTA coated and non-EDTA vials for analyzing the hematological parameters and serum biochemistry. Red blood cells (RBCs) count, total and differential white blood cells (WBCs) count, platelet levels and hemoglobin (Hb) content were determined using hematology analyzer (Model-Diatron, Wein, Austria).

#### 2.3.4 Biochemical analysis

Serum was separated by centrifuging at 5000 rpm for 10 min at 4 °C and was stored in a clean sample bottle at −20◦C for further analysis. The total bilirubin was determined as detailed by the Pearlman method [28]; alkaline phosphatase (ALP) was estimated by p-nitrophenyl picolinate (PNNP) hydrolysis; alanine aminotransferase (ALT) and aspartate aminotransferase (AST) were estimated using kinetic method kits supplied by M/s Raichem, India, using a Micro lab 300 auto-analyzer (Merck, Mumbai, India); the total protein concentration was determined by the Biuret method [29]. Kidney function marker, such as creatinine, were estimated by Jaffe’s kinetic [30]. The total cholesterol was estimated by the CHOD–PAP (cholesterol oxidase-phenol + amino phenazone) enzymatic method [31]; triglycerides by the GPO–PAP (glycerol-3-phosphate oxidase — phenol + amino phenazone) method [32]. HDL cholesterol by precipitation with phosphotungistic acid. LDL cholesterol by the equation LDL = total cholesterol − (HDL + VLDL). Serum sodium, and potassium were estimated using a flame photometer with an ion selective electrolyte analyzer. Chloride was estimated by the mercurous thiocyanate method using a kit from M/s Raichem, India.

#### 2.3.5 Organ weights and histopathological studies

At termination of treatment period (day 90) and at the end of recovery period (day 118), animals were sacrificed and complete necropsies were carried out. Necropsy was performed to analyze the macroscopic external features of the organs. The weight of liver, kidney, heart and spleen were recorded and expressed in relation to the final body weight. Histophatological investigation was done according to method of Abd-elhamid et al [33]. The tissue samples were fixed in 10% formalin, subjected to dehydration process, embedded in paraffin and were sectioned into slices of 2 μm followed by hematoxylin –eosin (E&H) staining for histopathological examinations. The pathological observation of all tissues were performed on gross and microscopic bases using an optical microscope of 100 × magnifications (Olympus-Magnustrinocular microscope, Tokyo, Japan).

#### 2.3.6 Urinalysis

The experiment was performed as per the method described in Liju et al., [34]. Briefly, five male and five female rats were orally administered with single dose of FHE at 1000 mg/kg b.wt and were transferred to metabolic cages (one animal/cage). After 24 h, urine samples were collected. During the study time, the animals were deprived of food, but provided with water *ad libitum*. The pH and volume of collected urine was measured. Glucose and albumin content of the urine was analysed using Magistik-GP urinalysis strip. Microscopic evaluations of the sediments was carried out to detect calcium or phosphate crystals.

#### 2.3.7 Opthalmic observations

Opthalmoscopic observations were performed as per the method described in Liju et al., [34]. Briefly, five male and five female rats were orally administered with single dose of FHE at 1000 mg/kg b.wt. After 24 h, ophthalmic examinations of the anterior portion of the eye dilated with a mydriatic agent was performed using an ophthalmoscope, in presence of a veterinary doctor.

### 2.4 Genotoxicity studies

#### 2.4.1 Mutagenicity assay

Evaluation of the ability of FHE to induce reverse mutation at the histidine loci of various *Salmonella typhimurium* strains TA98, TA 100 and TA 102 (Ames test) was conducted according to the standard procedures [35,36]. Mutagenicity of FHE was done by plate incorporation method in the presence and absence of an exogenous metabolic activation system at four doses (0.5, 1, 2.5, 5 mg/plate), in triplicates for each dose. 2.5 μg sodium azide/plate dissolved in distilled sterile water was used as positive control. A plate without drug and mutagens was used as negative control and 200 μL DMSO was used as the vehicle control. In the case of S9 mix activated group, acetamidofluorene (20 μg) was used as positive control. 2 mL top agar layer (0.6% agar and 0.5% NaCl) containing *S. typhimurium* strains, 0.5 mM histidine–biotin solution and different concentrations of FHE were shaken well and poured onto 25 mL of agar. The plates (triplicate) were incubated for 48 h at 37 °C, and revertant colonies were counted using a colony counter. Rat liver microsomal enzyme was used for metabolic activation of mutagen *in vitro* [37]. Microsome P450 enzymes was induced in rat liver by oral administration of 0.1% phenobarbital dissolved in water for 4 days. The animals were sacrificed on the 5th day and the liver were excised aseptically and microsomal S9 fraction was prepared by centrifuging the homogenate at 9000 g for 15 min. Activation mixture was prepared by mixing S9 mix (500 μL) with sodium phosphate buffer (0.2 M, pH 7.4), NADP (0.1 M), glucose-6-phosphate (1 M, pH 7.4), MgCl_2_–KCl (10 μL) in presence of mutagen, 2-acetamidoflourene (20 μg/plate) or different concentrations of FHE and bacterial strains TA 98 and TA 100. The fractions were incubated at 37 °C for 45 min. Further, it was mixed with 2 mL of molten top agar supplemented with histidine and biotin (0.05 mM). The mixture was shaken well and poured onto the surface of 25 mL of minimal agar. After 48 h incubation, the mutagenic response was evaluated by counting the revertant colonies per plate and comparing with the control groups. The test substance was considered to be mutagenic if there was a three- fold increase in the tester strains when compared to the negative control.

### 2.5 Statistical analysis

The values were expressed as mean ± SD. The data on body weight, food intake, organ weights, haematology and clinical chemistry were subjected GraphPad Prism analysis employing Version 5.00, USA. 2007. Independent sample *t*-test was used for analysing the statistical significance in trend of body weight and the differences between the groups were considered to be significant when p < 0.05. SPSS Software Version 22.0 was used for the analysis. The statistical significance was compared between control and experimental groups by one way analysis of variance (ANOVA) followed by appropriate posthoc test (Dunnet multiple comparison test). Data of FHE treated animals were compared with that of control animals, and the differences between the groups were considered to be significant when p < 0.05.

## 3 Results

### 3.1 Oral acute toxicity study (14 days)

Oral administration of FHE at 500, 1000 and 2500 mg/kg body weight did not produce any mortality or adverse effects during the 14 days period of study with no abnormal clinical signs. The oral LD_50_ of FHE in Wistar rats was found to be greater than 2500 mg/kg b. wt. /day. Feed and water intake and body weight remained in the normal range as compared to the normal control group (data not shown). Necropsy at the end of study did not reveal any gross pathological abnormalities suggesting the safety of FHE at the tested dose of 2500 mg/kg/day for rats.

### 3.2 Subchronic study (90 days) and reversal 28 days

All animals in the study groups survived until the scheduled necropsy on day 91 and the recovery group animals also survived till the end of study (118^th^ day). No mortalities were observed in the FHE treated groups of both sexes, at doses of 250, 500 and 1000 mg/kg b. wt. No treatment related abnormalities in clinical and/or behavioral signs were also observed as compared to the untreated control group of rats.

#### 3.2.1 Effect on body weight

**Fig. 1:**
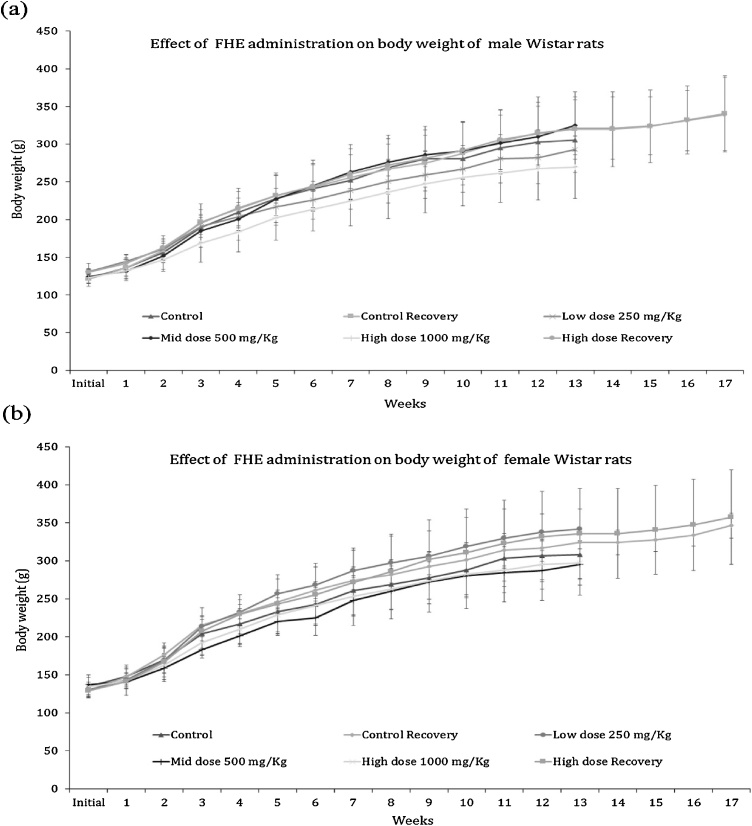
Effect of oral administration of FHE on the body weight of (a) male and (b) female rats during 90-days repeated dose toxicity study. Data are expressed as mean ± (SD) and analyzed by two-way ANOVA, as compared to respective parameter value of vehicle control (VC).

The weight gain among the male and female animals in the untreated and treated groups was normal (p > 0.05) (Fig. 1a). During 90 days study period, the body weight (g) of untreated control male animals increased from 122.4 ± 6.2 to 305.2 ± 19.2 with an average growth rate of 2.03 ± 0.14 g/day and that of female animals increased from 134.6 ± 12 to 308.2 ± 32.6 with a growth rate of 1.93 ± 0.23 g/day. Post 28 days observation also showed no significant effect on the recovery control group among both male and female rats; body weight increased from 121.4 ± 5.4 to 340.8 ± 49.9 and 128.8 ± 9.3 to 346.4 ± 16.6 respectively. FHE treated (250, 500 and 1000 mg/kg b. wt.) animals also showed no significant difference in the weight gain and growth rate among the male and female rats group when compared to the untreated control group of animals. The body weight (g) of male rats administered with high dose (1000 mg/kg b. wt.) of FHE was found to be increased from 123 ± 11.4 to 269.4 ± 41.2 with a growth rate of 1.63 ± 0.33 g/day and that of female rats increased from 134.4 ± 6.4 to 297.4 ± 28.8 with a growth rate of 1.81 ± 0.25 g/day. A similar trend was observed with the lower doses of FHE as well (250 and 500 g/kg b. wt.). The high dose recovery group IV also showed no significant effect in their body weight among male and female rats group; the body weight increased from 130.4 ± 11.4 to 339.2 ± 49.4 and 129.2 ± 7.6 to 357.4 ± 62.3 respectively (Fig. 1b).

Even though the body weight for the treated groups II (low dose, male) and IV (high dose, male) were lower than the control group at the end of the study, inter group comparison of male rat body weight using independent sample *t*-test showed no significant difference when compared to control (p > 0.05; CI = 95%). Similarly, female body weight comparison also showed no significant change in control recovery, low dose, mid dose, high dose and high dose recovery when compared to the control (p > 0.05; CI = 95%).

#### 3.2.2 Effect on food consumption

**Fig. 2:**
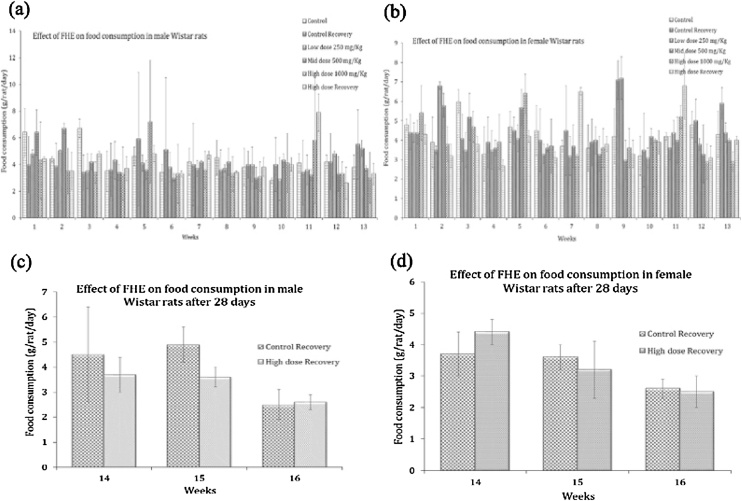
Food intake pattern of (a) male (b) female rats during 90-days of FHE administration. (c) and (d) represents the food consumption during 28-days of recovery period for male and female rats respectively.

Administration of FHE at 250 mg/kg (low dose), 500 mg/kg (mid dose) and 1000 mg/kg (high dose) did not produce any significant difference (p > 0.05) in the food consumption of male and female rats when compared to untreated group of animals (Fig. 2(a & b)). The average food intake of male rats was nearly 3 ± 1.9 g/animal/day and that of female was 2.9 ± 0.5 g/animal/day. High dose recovery group IV also showed no significant difference in the food consumption among the male and female rats 2.6 ± 0.3 and 2.5 ± 0.5 respectively when compared to the untreated group of male and female rats as 2.5 ± 2.1 and 2.6 ± 0.3 respectively [Fig. 2(c & d)]. Water consumption of the FHE treated animals also remained unchanged when compared with untreated control animals.

#### 3.2.3 Urine analysis

There were no significant changes in either the pH (6.5–7.5) or in the volume of urine collected from the FHE treated animals at various dosages (250, 500 and 1000 mg/kg b.wt) when compared with untreated. Microscopy of the urinary sediment did not reveal any calcium or phosphate crystals. Urinary glucose, albumin and keto acids were also absent in both treated and untreated animals.

#### 3.2.4 Ophthalmic observations

Ophthalmoscopic observations did not reveal any treatment related changes to conclude corneal ulcer or retinal vascularity. There was neither compression of retinal vessels nor extra branching of vessels, ruling out the plausible lesions like glaucoma or intraocular inflammatory changes due to FHE administration even at the highest dose of 1000 mg/kg b. wt. used in the present study.

#### 3.2.5 Necropsy and organ weights

Necropsy of the FHE treated animals showed normal appearance of various organs and tissues. The weight of organs (heart, liver, spleen and kidney) relative to the body weight showed no significant changes (p > 0.05) among the various treated animals when compared with the untreated control group of male and female animals (Table 1).

**Table 1:** Effect on administration of FHE on organ weight.

	Liver	Kidney	Heart	Spleen
MALE				
Control	9.4 ± 0.5	2.1 ± 0.2	1.1 ± 0.1	9.4 ± 0.5
250 mg/Kg	7.7 ± 0.9	1.6 ± 0.4	0.9 ± 0.2	7.7 ± 0.9
500 mg/Kg	8.5 ± 1.0	1.9 ± 0.3	0.9 ± 0.1	8.5 ± 1.0
1000 mg/Kg	8.8 ± 0.4	1.7 ± 0.1	1.0 ± 0.1	7.8 ± 0.1
Control Recovery	6.8 ± 0.3	1.4 ± 0.1	0.9 ± 0.1	0.9 ± 0.1
High dose Recovery	7.1 ± 0.4	1.5 ± 0.2	0.9 ± 0.1	0.9 ± 0.1
FEMALE				
Control	9.5 ± 2.2	2.1 ± 0.6	1.0 ± 0.2	9.5 ± 2.2
250 mg/Kg	9.4 ± 0.6	2.0 ± 0.2	1.0 ± 0.1	9.4 ± 0.6
500 mg/Kg	9.7 ± 0.9	1.9 ± 0.1	1.0 ± 0.1	9.7 ± 0.9
1000 mg/Kg	7.6 ± 1.0	1.6 ± 0.2	0.9 ± 0.1	0.9 ± 0.2
Control Recovery	7.0 ± 0.4	1.5 ± 0.1	0.8 ± 0.0	0.9 ± 0.2
High dose Recovery	6.7 ± 10.1	1.4 ± 0.1	0.8 ± 0.1	0.9 ± 0.2

Values are mean ± standard deviation, expressed as the organ weight/100 g of body weight. 5 animals/sex/group, unless otherwise specified.

#### 3.2.6 Hematological parameters

FHE did not produce any significant (p > 0.05) changes in the hematological parameters. Hemoglobin, WBC, RBC, platelet counts and differential counts (lymphocyte, eosinophil and neutrophils) of FHE treated animals remained in the normal range, when compared to the untreated control group of animals (Table 2). Similarly in the high dose (1000 mg/kg/day) recovery group animals (male/female), no significant alterations in the hematology parameters were observed. There were no statistically significant differences when compared with control and treatment groups. These results demonstrate that administration of FHE to rats at doses up to 1000 mg/kg/day had no adverse hematological effects.

**Table 2:** Effect of administration of FHE on hematological parameters.

	Control	Control Recovery	250 mg/Kg	500 mg/Kg	1000 mg/Kg	High dose Recovery
MALE						
RBC (10^6^ cells/μL)	8.6 ± 4.5	6.8 ± 0.6	11 ± 5.1	10.3 ± 4.7	12.1 ± 4.2	13.5 ± 3.9
WBC (10^3^ cells/μL)	14.3 ± 2.6	11.2 ± 4.1	11.8 ± 3.8	13.5 ± 3.8	12.5 ± 3.4	13 ± 2.7
Hb (g/dL)	12.2 ± 0.8	12.7 ± 1.1	12.8 ± 0.8	12.3 ± 0.5	12.4 ± 0.8	11.9 ± 0.4
Platelet (10^3^ cells/μL)	674 ± 132.7	631.8 ± 163.5	690.6 ± 114.4	639.6 ± 118	668.2 ± 193.4	641.6 ± 133.2
FEMALE						
RBC (10^6^ cells/μL)	9.8 ± 3.4	9.5 ± 4.2	10.8 ± 4.4	8.1 ± 1.2	9.6 ± 3.9	10.6 ± 5.5
WBC (10^3^ cells/μL)	12.6 ± 2.9	11.7 ± 3.3	14.8 ± 1.2	11.9 ± 2.8	12.9 ± 2.5	14.1 ± 1.9
Hb (g/dL)	12.5 ± 1	12.6 ± 1.1	13.5 ± 1.1	12.7 ± 1.1	13.1 ± 0.5	12.4 ± 0.6
Platelet (10^3^ cells/μL)	586.6 ± 148.4	629.2 ± 108.9	595 ± 127.2	496.8 ± 154.3	642.6 ± 146.9	661.8 ± 105.7

Abbreviations: Hb hemoglobin; WBC white blood cells; RBC red blood cells. The values are expressed as mean ± standard deviation. 5 animals/sex/group unless otherwise specified.

#### 3.2.7 Serum biochemical parameters

FHE administration did not produce any significant changes on biochemical parameters related to hepatic and renal function as compared to the untreated control group of animals. Renal function parameters such as serum creatinine and electrolytes in both male and female rats were comparable to the untreated control rats after 90 days of supplementation (Table 3). Hepatic function markers such as ALT, AST, ALP and total protein were not altered in FHE treated rats of both sexes (Table 3). Lipid profile also remained unchanged with no significant (p > 0.05) variation in total cholesterol, HDL and LDL cholesterol levels among both male and female rats, and were comparable to that of untreated control group (Table 4). However, the values were within normal laboratory ranges.

**Table 3:** Effect of administration of FHE on hepatic function markers and renal function parameters.

	Control	Control Recovery	250 mg/Kg	500 mg/Kg	1000 mg/Kg	High dose Recovery
MALE						
ALT (U/L)	63.5 ± 26.9	71.6 ± 16.9	72 ± 14.8	64.1 ± 24	66.9 ± 16.6	40.6 ± 2.3
AST (U/L)	142 ± 25.2	160.4 ± 22.5	147.6 ± 16.1	138.4 ± 19.4	135.8 ± 24.6	156.2 ± 25.4
ALP (U/L)	117.9 ± 34.9	112.8 ± 42.9	104.9 ± 25.5	111.5 ± 39.8	144 ± 55.6	112.3 ± 13.9
TP (mg/dl)	7.6 ± 0.4	8.3 ± 0.8	7.9 ± 0.6	8.1 ± 0.5	8 ± 0.6	7.7 ± 0.2
Creatinine (mg/dl)	0.9 ± 0.1	0.9 ± 0	0.9 ± 0.1	0.9 ± 0.1	0.8 ± 0.1	0.9 ± 0
Na (mmol/L)	134.6 ± 4.9	134.4 ± 4.3	135.8 ± 5.9	135.8 ± 3.2	133.6 ± 4	135.6 ± 2
K (mmol/L)	3.5 ± 0.6	3.9 ± 0.4	3.7 ± 0.6	4.1 ± 0.6	4.3 ± 0.3	4.3 ± 0.2
Cl (mmol/L)	112.3 ± 2.3	105 ± 3.5	100 ± 6.1	96.5 ± 2.2	98.5 ± 4.4	101 ± 4.5
FEMALE						
ALT (U/L)	55.3 ± 7.3	82.4 ± 24.8	59.7 ± 12	66.4 ± 17.3	50.7 ± 12.8	69.1 ± 12.6
AST (U/L)	155.4 ± 20.1	141.2 ± 19.4	150.6 ± 15	147.2 ± 37.1	155.2 ± 25.8	143.8 ± 26.4
ALP (U/L)	104.6 ± 60.3	97 ± 60.5	93.5 ± 33.3	98.4 ± 49.8	127.7 ± 42.2	121 ± 33.4
TP (mg/dl)	7.9 ± 0.4	7.8 ± 0.5	8.2 ± 0.2	8.2 ± 0.7	7.8 ± 0.4	8.4 ± 0.5
Creatinine (mg/dl)	0.8 ± 0.1	0.8 ± 0.1	0.9 ± 0.1	0.9 ± 0	0.9 ± 0	0.9 ± 0.1
Na (mmol/L)	133.4 ± 3.1	132 ± 4	137.4 ± 3.3	137.4 ± 2.8	132 ± 5.2	136.8 ± 5.4
K (mmol/L)	3.9 ± 0.2	4 ± 0.1	3.9 ± 0.3	3.7 ± 0.8	3.4 ± 0.7	3.8 ± 0.4
Cl (mmol/L)	103.2 ± 4.3	98.5 ± 4.2	95.6 ± 3.5	96.6 ± 6.5	98.3 ± 3.5	98.5 ± 2.4

Abbreviations: ALT, alanine aminotransferase; AST, aspartate aminotransferase; ALP, alkaline phosphatase; TP, total protein; Na, sodium; K, potassium; Cl, chloride. The values are expressed as mean ± standard deviation. 5 animals/sex/group, unless otherwise specified.

**Table 4:** Effect of administration of FHE on lipid profile.

	Control	Control Recovery	250 mg/Kg	500 mg/Kg	1000 mg/Kg	High dose Recovery
MALE						
Cholesterol (mg/dL)	46.3 ± 9.3	58.2 ± 15.3	50 ± 24.7	51.7 ± 7.5	51.8 ± 10.9	57.7 ± 16
Triglycerides (mg/dL)	61.6 ± 38.3	54.4 ± 11	55.3 ± 14.7	52.1 ± 23.2	49.6 ± 8.2	59.4 ± 13.2
HDL (mg/dL)	22.8 ± 4.2	30.8 ± 3.1	27.4 ± 7.9	26.7 ± 4.6	26.3 ± 3.8	25.5 ± 7.1
LDL (mg/dL)	10.9 ± 2.7	14.1 ± 10.8	16.9 ± 16.9	9.8 ± 2.9	14.4 ± 8.8	10.5 ± 3.3
FEMALE						
Cholesterol (mg/dL)	52.3 ± 6.8	55.8 ± 13	52.3 ± 16.1	64.9 ± 17.2	65.9 ± 4.6	66.1 ± 9.6
Triglycerides (mg/dL)	60.7 ± 21.8	51 ± 18.3	40.4 ± 12.7	51.9 ± 13.1	67.2 ± 24	51.8 ± 16.7
HDL (mg/dL)	27.7 ± 4.9	30.8 ± 3.3	29.1 ± 4.6	30.8 ± 4.6	28.6 ± 1.3	33.6 ± 5.9
LDL (mg/dL)	17.1 ± 5.4	16.7 ± 7.5	16.5 ± 9.1	25.8 ± 9.4	23.3 ± 11.3	22.9 ± 8.9

Abbreviations: HDL, high density lipoprotein; LDL, low density lipoprotein. The values are expressed as mean ± standard deviation. 5 animals/sex/group, unless otherwise specified.

The results of serum biochemical analysis in treatment and recovery group animals showed no toxicologically significant adverse effects in rats even after continuous administration of FHE at doses up to 1000 mg/kg/day for 90 days.

#### 3.2.8 Histopathological analysis

**Fig. 3:**
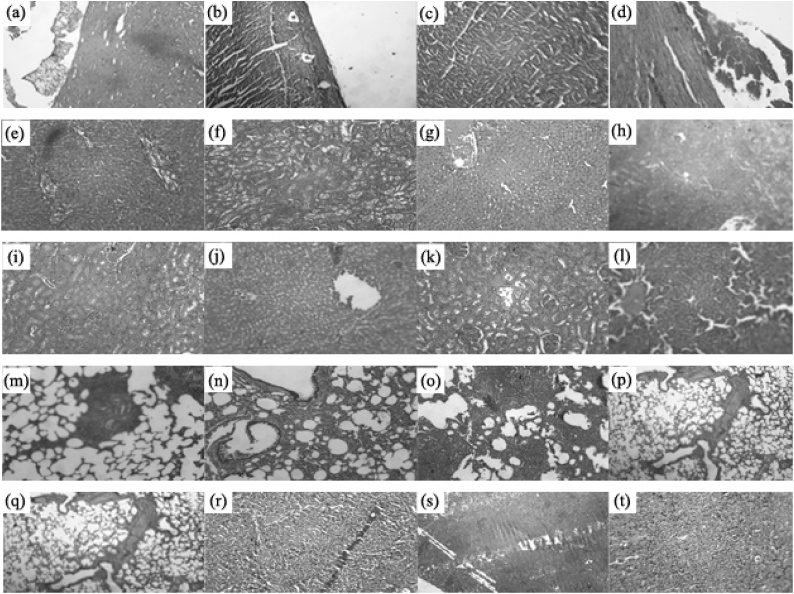
Effect of FHE on the histological examination heart (a–d), liver (e–h), kidney (i–l) lung (m–p) and spleen (q–t) tissues of rats during 90-days repeated of toxicity study. Photomicrographs a, e, i, m, q represents the control group; b, f, j, n, r represents the control recovery group; c, g, k, o, s represents the high dose group (1000 mg/kg b. wt.); d, h, l, p, t represents the high dose recovery group (1000 mg/kg b. wt.).

The histopathological examination of various organs of animals treated with 1000 mg/kg b. wt. of FHE showed normal cellular architecture when compared with those of the untreated groups of animals (Fig. 3). The tissue sections of spleen from FHE treated animals showed normal lymphoid follicles with areas prominent in germinal centers. Medullary region showed sinusoidal congestion, lymphostasis and histiocytic proliferation with cellular architecture and morphology similar to that of untreated control animals. The liver section of FHE treated animals showed normal portal triads and central venous system; normal hepatocytes were arranged in cords with Kupffer cells and showed normal sinusoidal spaces, which were identical with those from the untreated animals. The histopathology of kidney tissues of FHE treated animals showed normal glomeruli with Bowman’s capsule adrenal tubules. The interstitial tissues (lungs) appeared with no apparent abnormalities when compared with the tissues of untreated group of animals. The histopathology of heart in both treated and untreated group of animals showed normal myocardial fibers in longitudinal section featuring central nuclei and syncytial arrangement of fibers.

#### 3.2.9 Mutagenicity of FHE

FHE did not show any substantial increase in revertants in any of the strains of *S. typhimurium* when used either in the presence or in the absence of metabolic activation (S9 mix) (Table 5). While positive controls containing the known mutagens resulted in a significant (p < 0.05) increase in the revertant colonies in each strain, vehicle control did not produce any change. The spontaneous reversion rates in the negative and positive control were within the normal range which suggested that FHE did not induce any gene mutation or frame shifts in the genome of the strains under the specific conditions of the experiment.

**Table 5:** Mutagenic study of FHE on *S. typhimurium* strains using reverse mutation assay.

Doses of FHE (mg/plate)	TA 98	TA 100	TA102
*Without S9-mix*	*Number of revertants*		
Negative control^b^	61 ± 5.8	78 ± 2.4	71 ± 5.8
Positive control^c^	410 ± 31	770 ± 75	918 ± 70
5	55 ± 3.9	76 ± 5.5	68 ± 3.7
2.5	58 ± 4.8	69 ± 7.4	70 ± 5.3
1	65 ± 7.5	73 ± 9.8	73 ± 7.2
0.5	56 ± 5.1	77 ± 6.1	66 ± 8.6
*With S9-mix*	*Number of revertants*		
Negative control^b^	67 ± 3.1	87 ± 6.7	106 ± 15.1
Positive control^c^	524 ± 32	645 ± 56	467 ± 39
5	65 ± 5.3	62 ± 7.5	102 ± 16.8
2.5	61 ± 7.9	81 ± 5.9	110 ± 11.8
1	68 ± 6.3	76 ± 8.3	109 ± 12.5
0.5	64 ± 8.5	85 ± 14.2	104 ± 10.2

The values are mean ± SD of 3 different determinations. Acetamidofluorene was used as mutagen in studies involving S9 activities. **^b^** Spontaneous reversion (without mutagen and drug). **^c^** Mutagen treated (NPD for TA 98; NaN_3_ for TA 100 and TA 102).

## 4 Discussion

The present study investigated the safety of a standardized hydro-ethanolic extract of fenugreek seed husks (FHE) that has been shown to be biologically active and provided clinically significant outcome in the reduction of postmenopausal discomforts when supplemented at (500 mg × 2) /day for 8–12 weeks [26]. FHE was also shown to enhance the estradiol levels and helping to establish hormone balance by maintaining a healthy calcium and hemoglobin levels in postmenopausal women [26]. Thus, the significant biological activity of FHE warrants a systematic investigation on the plausible toxicity effects even though fenugreek seeds and its solvent extracted oleoresin have already given the status of GRAS for use as a ‘natural food flavor’ by US FDA [8]. Usually, the flavor dosage will be 0.1 to 0.3% (w/w). However, FHE has not revealed any adverse events or changes in the haematological/biochemical parameters when supplemented at 1 g/day level for 12 weeks to postmenopausal women [26].

FHE was shown to contain the most widely studied fenugreek phytochemicals, including the furostanic saponin ‘protodioscin’, the alkaloid ‘trigonellin’ and the amino acid 4-hydroxyisoleucine. FHE was prepared with an extract ratio of 25 to 30:1 (fenugreek seeds: FHE), which corresponds to the traditional usage level of 20–25 g seeds/day for various health related concerns among women. Various extracts of fenugreek seeds with varying compositions of phytonutrients have been investigated for toxicity. Ketan et al reported that single dose oral administration of 2 g/kg b. wt. of a protodioscin – rich extract (26% of protodioscin) does not produce any adverse events during 14 days of study period. The extract was also shown to be safe with no obvious acute toxicity when intravenously administrated at 1 g/kg b.wt. [23]. Yet another study demonstrated the safety of a single dose oral administration of fenugreek seed extract at 8 g/kg b. wt. without any sign of toxicity or mortality [24]. An ethanolic extract of fenugreek seeds showed no mortality and adverse effects when orally supplemented as a single dose of 3 g/kg b.wt. [25]. However, another fenugreek seed extract rich in the glycosides (trigonosides and vicenin) was shown to exhibit 40% mortality in experimental mice during oral acute toxicity study for 14 days at 5000 mg/kg b.wt. [38].

In the present acute toxicity study (14 days), FHE was found to be well abided by the experimental animals even at high dosage of 2500 mg/kg b. wt. The absence of mortality or abnormalities or adverse events during 14 days of observation period indicated its primary safety. Organization for Economic Corporation and Development (OECD) guidelines for oral acute toxicity has classified LD_50_ of 2 g/kg b. wt. as relatively safe, if did not produce any sign of toxicity, changes in behavior or death in the experimental animals [39]. Upon repeated dose for 90 days, FHE did not produce any mortality, adverse effects, clinical or behavioral symptoms even at 1000 mg/kg b.wt. Food and water consumption, and rate of increase in body weight were also not significantly different from the normal control group, indicating its safety. Changes in body weight and food/water consumption have generally been regarded as a preliminary indication of adverse effect of a drug [40].

The fact that all the animals (male and female) survived until the scheduled necropsy on 91^st^ day and for further 28 days in high dose recovery group indicated the primary safety of FHE in experimental rats. Further analysis indicated no significant changes in the hematology parameters (hemoglobin, RBC count, platelet count, total and differential leukocytes count), as compared to the untreated control group of animals, indicating the stability of hematopoietic system which was generally considered as one of the most sensitive set of parameters to assess the safety of a drug [41]. The chronic administration of FHE was also found to induce no significant changes in liver function markers. Liver function markers are very sensitive parameters of toxicity. For instance, elevation in serum ALT levels was usually regarded as the first response of liver cell damage [42]. Yet another important marker of liver health is the variations in lipid profile, since liver is the site for cholesterol degradation and glucose synthesis [43]. Treatment with FHE for 90 days demonstrated no changes in LDL, HDL and VLDL level with a non-significant reduction in LDL and triglyceride levels, which indicated the normal lipid and carbohydrate metabolism of FHE treated animals. Similarly, serum creatinine and electrolytes (sodium, potassium, chlorine and bicarbonate) levels also remained in the normal range indicating the safety of FHE on renal functions. An increase in the serum creatinine levels generally correlated to the damage of functional nephrons [44]. Kidney is a sensitive organ, whose function is known to be affected by a number of factors including phytochemicals and synthetic drugs, and may ultimately lead to renal failure [45]. Moreover, the absence of either the weight changes or morphological abnormalities of vital organs, as revealed by the macroscopic and histopathological investigations, also pointed towards the safety of FHE even upon repeated dose consumption for 90 days. But, the lack of blood glucose measurements, prothrombine time, and urea remain as a limitation of the present study.

The high dose recovery group also showed no significant changes in comparison to the control group indicating the safety and normal behavior of the animals even after withdrawal of the supplementation. The aims of observations on the recovery group were to see any delayed effects or recovery effect after administration of the test substance. Though there were no significant changes in animal behavior, food and water consumptions, and decrease in body weight in FHE treated group at any dosage, high dose recovery group (1000 mg/kg b. wt.) showed a trend towards the body weight increase after stopping the treatment. In sum, the subchronic study indicates that FHE ingestion did not induce detrimental change and morphological alterations in vital organs and has a “no observed adverse effect level” (NOAEL) of 1000 mg/kg/day. Though the information will help for future clinical studies on the medicinal and nutritional potentialities of FHE as a phytomedicine and/or phytonutrient, it should be emphasized that NOAEL may not always be entirely extrapolated to the safety in humans. However, an early investigation on postmenopausal women at (500 mg × 2)/day for 90 days does not produced any adverse event.

Ames test is considered as one of the most consistent test for detecting genotoxic and carcinogenic substances and was employed for evaluating the mutagenicity activity of FHE [36]. Upon Ames test, FHE did not produce any revertants at tested concentrations of 0.1–5 mg/plate, indicating the absence of dose related mutagenicity of FHE either with or without metabolic activation in the tested strains TA98, TA100 and TA102.

## 5 Conclusion

The present study reported the toxicological evaluation of a saponin-rich standardized extract of fenugreek seeds (FHE) as shown by the acute (14 days), subchronic (90 days) oral gavage at 1000 mg/kg b. wt. and mutagenicity studies. FHE did not produce any significant changes in body weight, food and water consumption, hematological or biochemical parameters. The lack of significant changes in both physical appearance and behavioral patterns further revealed the absence of treatment-related adverse effects of FHE. Histological examinations of selected organs also supported the safety of FHE. It did not produce any mutagenicity to Salmonella strains with and without activation of S9 mixture up to a concentration of 5 mg/plate. Thus, the results of the present study indicated that FHE is safe in rats with an NOAEL of 1000 mg/kg body weight per day and can be further considered for human studies. However, more robust studies with large number of animals on reproductive toxicity and genotoxicity study using more number of strains in GLP (good laboratory practices)-certified laboratories would add more reliable safety informations on fenugreek extracts.

## Conflict of interest

The author(s) declared no conflicts of interest with respect to the authorship and/or publication of the article.
